# Editorial: Reproduction and the Inflammatory Response

**DOI:** 10.3389/fendo.2021.835854

**Published:** 2022-01-31

**Authors:** Yang Yu, Hsun-Ming Chang, John Even Schjenken

**Affiliations:** ^1^ Department of Gynecology and Obstetrics, Key Laboratory for Major Obstetric Diseases of Guangdong Province, Key Laboratory of Reproduction and Genetics of Guangdong Higher Education Institutes, The Third Affiliated Hospital of Guangzhou Medical University, Guangzhou, China; ^2^ Beijing Key Laboratory of Reproductive Endocrinology and Assisted Reproductive Technology and Key Laboratory of Assisted Reproduction, Ministry of Education, Center of Reproductive Medicine, Department of Obstetrics and Gynecology, Peking University Third Hospital, Beijing, China; ^3^ Clinical Stem Cell Research Center, Peking University Third Hospital, Beijing, China; ^4^ Department of Obstetrics and Gynaecology, British Columbia Children’s Hospital Research Institute, University of British Columbia, Vancouver, BC, Canada; ^5^ Priority Research Centre for Reproductive Science, School of Environmental and Life Sciences, Discipline of Biological Sciences, The University of Newcastle, Callaghan, NSW, Australia; ^6^ Infertility and Reproduction Research Program, Hunter Medical Research Institute, New Lambton Heights, NSW, Australia

**Keywords:** endocrine disrupting compound (EDC), endometriosis, infection, inflammation, inflammasome, polycystic ovary syndrome (PCOS), preeclampsia, repeat implantation failure

Reproduction is a fundamental feature of all known life entailing a remarkably complex and tightly regulated series of events that are essential in supporting species’ long-term survival. While the development of knowledge of reproductive processes across species is of utmost importance, this Research Topic primarily focuses on those factors that influence the pathophysiology of reproductive complications or gynecological conditions in humans.

Unlike many other species, human reproduction is surprisingly inefficient. Infertility for example is a global public health issue rising in prevalence that affects upwards of 15% of reproductive-aged couples, or a total of 186 million individuals worldwide (1, 2). Even amongst those couples able to initiate pregnancy, challenges are still faced. Embryo loss between fertilization and birth is estimated between 40-60% (3), while pregnancy complications, including preeclampsia (PE, 2-8% of pregnancies) and preterm birth (10.6% of births), are the leading cause of death amongst women of reproductive years (4, 5). Gynecological conditions, such as endometriosis (10%) (6), polycystic ovary syndrome (PCOS, 5-20%) are common (7), in addition to sexually transmitted infections (Chlamydia, 2016 = 127 million estimated infections) (8) and parental lifestyle factors not only influence fertility but also lead to adverse birth outcomes and lay the foundations for offspring susceptibility to disease (9). While numerous contributing factors influence our susceptibility to these reproductive challenges, one area that is intimately involved in all areas of reproductive function and contributes to reproductive pathologies is inflammation and dysregulation of the immune system (10).

The immune system plays an integral role in both the physiology and pathophysiology of reproduction (10, 11). Across both males and females, there is a close functional relationship between the male and female reproductive tracts and the immune system, which is tightly regulated to meet the physiologically challenging demands of successful reproduction (12). In females, events including ovulation, menstruation, implantation, and parturition are all associated with the induction of inflammatory mediators, while in males, the development and function of testes and epididymis are influenced by their immune microenvironment (12, 13). Correct programming of the maternal immune environment supports implantation and subsequent pregnancy success (14). Thus, altered immune function during, or even prior to gestation can have a significant impact on not only our reproductive health, but also on the long-term health of offspring.

The reasons behind the decline in fertility and increased prevalence in subfertility are complex, but there is extensive evidence that lifestyle and environmental factors are major contributors (15). Endocrine Disrupting Compounds (EDCs) for example, influence reproductive function through their potential to mimic or block the actions of endogenous hormones (9). In this Research Topic, Schjenken et al., highlight the capacity of EDCs to also affect immune system development and function, and propose that in addition to disruptions in hormone signaling that regulate reproductive physiology, that alterations to the immune environment caused by EDC exposure during gestation may disrupt the establishment of maternal immune tolerance that is required to support robust placentation and fetal development (Schjenken et al.). Additionally, infection during pregnancy is also linked with adverse pregnancy outcomes. TORCH infections, which classically comprise toxoplasmosis, Treponema pallidum, rubella, cytomegalovirus, herpesvirus, hepatitis viruses, human immunodeficiency virus, and other infections, such as varicella, parvovirus B19, and enteroviruses are major contributors to prenatal and post-natal morbidity and mortality (16). However, most studies only focus on infections that occurs during pregnancy. In this Research Topic, Liu et al., explored the impact of previous TORCH infections on maternal and neonatal outcomes in an assisted reproduction setting and show that prior infections were not associated with adverse pregnancy or neonatal outcomes, thus highlighting that infections that occur during pregnancy should be the major focus of future study.

Alterations to the maternal immune environment are also implicated as a central predisposing factor in many of the common pregnancy complications, such as repeat implantation failure (RIF) and PE (17–19). Both conditions are commonly characterized by dysregulated activation of both innate and adaptive arms of the immune system (17, 19). In this Research Topic, Ho et al. explored how the proportion of peripheral natural killer cells influenced ART outcomes following intravenous immunoglobulin treatment while di Rivero Vaccari and Shirasuna et al. discussed recent reports that link pregnancy complications such as PE with altered inflammasome activation. In both complications, the authors show the potential benefit of developing a greater understanding of immune cell function in developing diagnostics or therapeutics to treat these pregnancy complications. In the case of RIF, intravenous immunoglobulin (IVIG) treatment is beneficial only in some patients, and Ho et al. demonstrated the prognostic capacity of assessment of peripheral populations of natural killer cells prior to conception. They showed that peripheral CD56+CD16+ NK cells with a population of ≤10.6% in RIF patients were likely to benefit from IVIG therapy with better implantation and pregnancy rate outcomes. In the case of PE, both di Rivero Vaccari and Shirasuna et al. highlighted the potential contribution of altered inflammasome activation to the pathophysiology of PE. Additionally, these reports highlight the therapeutic potential of inflammasome targeted treatments to treat reproductive associated problems, where inflammation and dysregulation of the maternal immune system is a contributing factor (di Rivero Vaccari, Shirasuna et al.)

Inflammation is also influential in the pathophysiology of common gynecological conditions such as endometriosis and PCOS. These conditions are characterized by chronic inflammation and alterations to immune cell phenotype and function. Endometriosis is characterized by the presence of endometrial cells outside of the uterine cavity that leads to symptoms including painful periods, chronic pelvic pain, and infertility (6, 20). A combination of factors, including dysregulation of the immune response and alterations in sex hormone signaling are thought to contribute to an inability to clear ectopic endometrium from the pelvic cavity (6, Garcia–Gómez et al., Hogg et al.). In this Research Topic, Garcia–Gómez et al., Hogg et al., and Borelli et al. all explored different aspects of the role of inflammation in the pathogenesis of endometriosis. Garcia–Gómez et al. highlighted the intimate association between hormones and inflammation in endometriosis. They showed that alterations to the cellular response to steroid hormones lead to dysregulation of the inflammasome pathway, with these changes contributing to disease progression through the prevention of cell death and promotion of adhesion, invasion, and cell proliferation (Borelli et al.). Hogg et al. provided an up–to–date review on the central role that macrophages play in the pathogenesis of endometriosis, focusing on their origins, phenotype, and function. They highlighted that modification of macrophage phenotype and function under disease–modifying conditions promote the growth, development, vascularization, and innervation of lesions in addition to the generation of pain symptoms (Hogg et al.). Finally, Borelli et al. explored the levels of mast cells in peritoneal fluid of women with endometriosis and examined whether dysregulation in the mast cell population may influence sperm function. They showed that mast cells and their main mediator tryptase are more represented in the peritoneal fluid of patients with endometriosis. Finally, using an *in vitro* model of mast cell–sperm interaction, the authors were unable to show a direct effect on sperm motility. However, incubation of the mast cell line, LAD2 with sperm and peritoneal fluid from patients with endometriosis did lead to a significant increase in the mast–cell degranulation response, which may influence sperm function (Borelli et al.).

PCOS is the most common endocrine disease and is a complex and heterogeneous disease characterized by a combination of signs and symptoms of 3 phenotypic characteristics: hyperandrogenism, ovulatory dysfunction, and polycystic ovarian morphology (7, 21). This condition is associated with various cardiometabolic risk factors, chronic inflammation, and an increased risk of infertility (22, 23). In this Research Topic, two manuscripts explored the association of PCOS with inflammation. In the first study, Ganie et al. explored biomarkers of inflammation in an Indian population of PCOS patients and compared dietary status to their inflammatory load. It is estimated that in India, 22.5% of reproductive–aged women have PCOS (24), and in this study, Ganie et al. demonstrated that in an Indian population, PCOS caused increases in serum pro–inflammatory cytokines (Tumor Necrosis Factor–alpha and Interleukin–6) and decreases in anti–inflammatory molecules (Interleukin–10 and Adiponectin) compared to healthy controls (23). In the other study, Cao et al. performed a retrospective study that explored the seasonal susceptibility of PCOS patients undergoing IVF treatments to ovarian hyperstimulation syndrome (OHSS), a severe complication of controlled ovarian hyperstimulation that is more common in patients with PCOS (25). Their data was used to generate a predictive model that showed that the incidence of patients with PCOS that were at high risk for OHSS was significantly higher during the winter and summer months in Henan province in China (Cao et al.).

Taken together, the papers that form this Research Topic present an overview of research surrounding the roles of inflammation in reproduction. In view of recent advances that define the pivotal role of inflammation in the normal physiology of reproduction and pathophysiology, there is a pressing need to determine how dysregulation of inflammation and immune responses contributes to pathologies that surround reproduction. Ultimately, improved mechanistic understanding of the immune contributions to reproductive success will identify novel targets for diagnostic and therapeutic intervention strategies aimed at alleviating the rising burden of infertility and subfertility.

## Author Contributions

YY, HC, and JS all contributed to compilation, drafting and writing of this editorial. HC and YY are considered equal first authors. All authors contributed to the article and approved the submitted version.

## Conflict of Interest

The authors declare that the research was conducted in the absence of any commercial or financial relationships that could be construed as a potential conflict of interest.

## Publisher’s Note

All claims expressed in this article are solely those of the authors and do not necessarily represent those of their affiliated organizations, or those of the publisher, the editors and the reviewers. Any product that may be evaluated in this article, or claim that may be made by its manufacturer, is not guaranteed or endorsed by the publisher.
